# Social capital, identification and support: Scope for integration

**DOI:** 10.1371/journal.pone.0266499

**Published:** 2022-04-14

**Authors:** Justin Richardson, Tom Postmes, Katherine Stroebe

**Affiliations:** Department of Social Psychology, University of Groningen, Groningen, The Netherlands; Shimonoseki City University, JAPAN

## Abstract

Social relationships are important predictors of a range of individual outcomes, such as wellbeing and health. These social relationships are conceptualised in different ways, such as (inter-personal) forms of *social support*, *identification* with groups, or *social capital*. What is the overlap among these concepts and in what ways do they differ? The present work aims to clarify this with empirical evidence from two panel studies (*N* = 3934; *N* = 2912). The studies include central measures of social relationships (group identification, group membership, social support and social capital). Empirical differences and overlap were studied by evaluating the factor structure of the data with both confirmatory factor analyses and bi-factor analyses. Results showed that the different concepts had a large amount of empirical overlap (together accounting for over 60% of common variance). Surprisingly, results also revealed that subcomponents were identifiable based on who they target and not based on their conceptualisation. For example, items about *identification with neighbourhood* factored together with *support* items from the *neighbourhood*, and not with other identification items. Accordingly, we conclude that in addition to a general factor, it is possible to meaningfully distinguish components of social relations based on which group is targeted by the items (e.g. neighbourhood or family and friends). For future research on the relationship between social relations and health, the present measures are unlikely to be sufficiently precise to disentangle whether health effects are caused by identification, support or capital. Differences between targets appear to be more important than differences between these concepts for understanding the relationship between social relations and health and wellbeing.

## Introduction

Social relations are essential for the functioning of society in general and for individual wellbeing in particular. Not only do social relations form an important part of every human being’s life, a large body of literature has acknowledged the benefits of having good social relations for individuals, including for outcomes such as health (e.g. [1]). The current paper deals with a deceptively simple question: what are "social relations"?

The concepts we study in the current paper, social support, multiple group memberships and social identification, and social capital, are central concepts in measuring social relations, yet they are only a few examples of the many ways in which this construct is often described and studied. From the large number of studies on social relations and their benefits we can safely say that this topic has the interest of a large community of researchers that stem from quite different scientific backgrounds such as clinical and social psychology, sociology and economics. Collectively they provide a large body of evidence showing that social relations are beneficial for humans in many ways, such as the afore mentioned health consequences, but also relating to for example political engagement [2] and occupational mobility [3, 4]. Most of the associations draw on the idea that the people around us, be it in the form of groups of people (e.g., neighbours, sport club) or individuals (e.g., a special person, a family member), provide us with help, knowledge or merely a feeling of embeddedness. However, the interpretation of these results is clouded by the variability in definitions and constructs that are being used in the literature, each of which point to slightly different aspects of relations that are "doing the work", so to speak.

The current work aims to shed light on the potential differences and commonalities between constructs that are considered theoretically central to the conceptualisation of social relations: social support, multiple group memberships and social identification, and social capital. These concepts partly stem from different research domains (i.e., clinical and social psychology, sociology and economics) and are rooted in three theoretical approaches: *social support*, *social identification*, and *social capital*. And though we acknowledge that many other conceptualisations of social relations exist, the three theoretical approaches studied in the current work stand out through their dominance in the literature. In two panel studies we test whether these concepts, in different constellations, can be empirically identified as separate entities (e.g., by performing confirmatory factor analyses) or whether they show empirical overlap and are better conceptualised by a general factor rather than as separate entities.

In the paper we first reflect on the characteristics of social relations that are (often implicitly) assumed to underlie these different concepts’ impact for health, such as the number of social relations (e.g. multiple group memberships [3]), levels of trust within social relations (e.g. social capital [4]), feelings of connectedness to different groups (e.g. social identification [5, 6]) or the help or support from others (e.g. social support [7]). In highlighting their impact on individuals, we shall primarily focus on how these concepts are supposed to affect health and wellbeing, because this domain contains several large literatures in which these concepts are central predictors of individual outcomes. We then empirically study how the operationalisations of these different concepts are related to one another. Our approach in doing so is stepwise across the studies: In first instance, in Study 1, we differentiate social support, multiple group memberships and social identification to address the conceptual overlap we identified between these approaches. The first study revealed only few traces of the original conceptual underpinnings of social support, multiple group memberships and social identification–and strong evidence for a more general overarching factor underlying these measures of social relations. Searching for what such an overarching concept might be, we then in Study 2 also included an existent broader conceptualisation of social relations: social capital. Would this concept better capture the essence of social relations? As Study 2 will illustrate, this is not the case: results again revealed a large amount of empirical overlap between the measures of social relations from Study 1 and a strong general factor underlying all concepts.

We hope that the insights from these studies will, in the short run, help researchers think about the conceptual underpinnings of their research and help them design research in such a way that it tests their hypotheses appropriately. In the long run, we hope that this approach shall provide researchers with a better insight into precisely which aspects of social relations benefit communities and individuals with respect to important outcomes such as health and wellbeing.

One of the most obvious consequences of social relations is that they provide individuals with networks of social support. *Social Support* is defined in many different ways, but most often involves the transaction between two or more individuals [8, 9]. This means that it is also measured in relation to individual people, such as a special person, friend, or family member. Generally speaking, social support via these different individuals, offers people something concrete that is beneficial to them. This can either take on the form of *instrumental support* (e.g. helping a neighbour do their groceries), *emotional support* (e.g. expressing empathy to someone in need) and/or *informational support* (e.g. providing someone with information enabling them to cope with their problems) [10, 11]. Definitions of social support tend to stress somewhat different elements as being essential to social support. For example, Shumaker and Brownell [9] elaborate on social support as being “an exchange of *resources* between at least two individuals perceived by the provider or recipient to be intended to enhance the wellbeing of the recipient” (p. 13). Cobb [12] describes social support as a signal that one is *cared for* and loved for, esteemed and a member of a beneficial network. According to Cobb, the individual benefits of such an environment go beyond the warmth and comfort of social cohesion: support protects people against many forms of health issues (e.g. depression, but also tuberculosis), through a process of coping.

Though definitions across studies tend to differ slightly, numerous studies have confirmed that social support is beneficial for wellbeing and health [8, 13–16]. Social support is thought to promote health through the following mechanisms: stress-buffering and direct health effects [13, 17]. Cohen and Wills set out substantial evidence for a buffering function, in which the impact of social support on health is particularly beneficial for those suffering adversities and stressful life events. This buffer consists of the ability of a person to access resources in their social network in times of stress. In other words, here social support is only beneficial in situations that one needs help. In contrast, the direct health effect of social support can be defined as the benefits one can derive from a social network, irrespective of an adverse situation. This is where social support has positive implications in itself. The direct beneficial health effects of social support stem, among others, from the health promoting effects of one’s social network via perceived norms in this network and subsequent social influence. For example, social norms against smoking within one’s network, can prevent individuals from smoking and as a consequence improve health.

*Social identification*, rooted in the Social Identity Approach (SIA), was developed on the basis of social identity [18] and self-categorisation theory [19, 20]. SIA is a social-psychological theory of social relationships grounded in a social model of self [21]. According to the SIA, people define themselves partly in terms of their personal, partly in terms of their social identity. People are thought to act in line with such ‘identities’, depending on which is made salient in a particular context. When identification is measured, it is measured by referring to a potential group people might affiliate with, such as other women, one’s sports club, neighbourhood etc.. Identifying with such a meaningful group means having a strong sense of "we-ness” with the group: when group members have a shared social identity and when this shared identity is salient, the distinctions between "me" and "you" are subsumed in an overarching sense of "us" that influences group members’ cognitions and behaviour [22]. Ultimately, according to the SIA, this shared social identity is at the core of every form of social behaviour. In the words of Turner [19]: “social identity is what makes group behaviour possible” (p. 21).

The literature on the relationship between social identity and health identifies four mechanisms that are thought to explain the positive relation between social identity and health: social identity as a determinant for symptom appraisal and responses, social identity as a determinant of norms and behaviour, social support as a consequence of shared social identity and social identity as a coping resource [22]. In the first mechanism, symptom appraisal and responses are moderated by salient social identifications. For example: People who think of themselves as ‘cold sufferers’ are more likely to report symptoms and seek medication for their cold [23]. The second, concerning norms and behaviour, explains people’s behaviour in certain contexts, based on the norms that are associated with one’s identities. For example, strong identifiers with an ethnic minority group, were more likely to actively oppose health-related messages from an outgroup [24]. The third mechanism relates to access to social support: social support is facilitated by a shared social identity and is seen as mediating the relation between identification and health [5, 25]. The fourth mechanism suggests that social identity itself can also be a coping resource. For instance, members of a stigmatised group may find that a shared social identity provides them not only with a social network for giving and receiving social support to cope with the *individual consequences* of stigma, but also with a shared understanding that allows them to *collectively* resist the stigma itself by, for example, challenging it (e.g., [26]).

The social identity approach also instigated research looking at the more quantitative side of social relations: Is it beneficial for health to be a member of many different groups [32]? The concept *of multiple group memberships*, as it is referred to, builds on the idea that group identification provides one with a resource in times of need. It is measured by asking whether one for example feels one is a member of many different groups. The main reason why the number of groups one identifies may benefit the individual is a straightforward summative one, not too dissimilar from the concept of social capital: the more groups one belongs to, the more likely one may benefit from one or multiple of them in times of need [3]. Illustrative of this, is a study by Iyer and colleagues (2009), showing the association between the number of groups first year university students belonged to, and the social support they received. As one scholar put it: “Psychologically speaking, having one’s eggs in multiple baskets enhances the likelihood of having some of those eggs intact after an accident” ([27], p. 675). Indeed, there is evidence that having multiple group memberships and maintaining them is related to wellbeing [27, 28]. Though multiple group membership is sometimes referred to and measured as the ‘actual number’ of groups one belongs to (for example in Cruwys et al. [29]), the current research uses an operationalisation that measures the *perception* of group membership. It is also in this operationalisation we find a distinction with the element of group membership included in social capital measures (on which we elaborate later on). Multiple group membership here, coming from a social identity approach, is theorised as the *perception* of belonging to one or more groups. Multiple group membership as it is used in the current research, goes beyond an absolute number and captures one’s judgement about what that person sees as ‘many’ groups (for example: people will vary in what they find ‘many’ groups).

Social relations are conceptualised more broadly in the *Social Capital* literature (for an overview see [30, 31]). In keeping with a more structural analysis of human networks and the benefits that such networks have for individual "nodes", Harpham, Grant and Thomas [17] define social capital as the degree of one’s connectedness, and the quality and quantity of one’s social relations. Putnam et al., [4] defined social capital as “features of social organization, such as trust, norms and networks, that can improve the efficacy of society by facilitating coordinated actions”. Social capital is often separated into two dimensions: *structural* social capital (what people do and how they behave within their accessible networks) and *cognitive* social capital (perceptions of trust, reciprocity and sense of belonging. At a theoretical level it thus encompasses elements that we find in other conceptualisations such as social support and multiple group membership, which both to some extent resemble structural social capital. Both social identification and cognitive social capital stress the importance of sense of belonging to a group. We note, as illustrated by Tables 1 and 3 that these concepts are operationalised in quite different ways (e.g., perceived support versus actual support for social support versus social capital respectively).

**Table 1:** Measures used in Study 1.

Scale name	Scale Items
Four-Item identification with **Neighbourhood/Village** scale^b^	1. I am glad to be part of my village or neighbourhood
	2. I feel committed to the people in my village or neighbourhood
	3. I identify with the people in my village or neighbourhood
	4. Being part of my village or neighbourhood is an important part of how I see myself.
Single-Item identification with **Neighbourhood/Village**^a^	I feel connected to the people in my village or neighbourhood
Four-Item identification with **Family/Friends** scale^b^	1. I am glad with my Family/Friends
	2. I feel committed to my Family/Friends
	3. I identify with my Family/Friends
	4. My family and friends are an important part of how I see myself.
Single-Item identification with **Family/Friends**^a^	I feel connected to my family/friends
Single-Item *Social support* from **Neighbourhood/Village**^a^	I receive the help and support from the people in my village or neighbourhood
Single-Item *Social support* from **Family/Friends**^a^	I receive the help and support from my family/friends
Multiple Group Memberships Scale^b^	1. I belong to lots of different groups.
	2. I am involved in the activities of lots of different groups.
	3. I have friends who are in lots of different groups.
	4. I have strong ties with lots of different groups.

^a^ Used in Study 1a, single items were scored on a 5-point scale 1 = “I totally disagree”, 5 = “I totally agree”

^b^ Used in Study 1b.

Underlying all conceptualisations and definitions of social capital is the idea that social relations are a resource, a form of capital akin to wealth or intelligence, available to communities or individuals. One could say that of all the concepts of social relations used in the current study, social capital tends to be the most ‘abstract’ level of measurement. Social capital is also defined as an inclusive measure that touches on core social structures, both horizontally and vertically (between individuals or groups, *and* institutions) [32, 33]. Its level of abstraction is also reflected in the operationalisation of social capital, which often includes measures of ‘civil engagement’ and ‘community trust’ relating to a wide range of communities such as religious, protest or political groups as well as neighbourhoods. Civil engagement and trust are elements we do not often find in the more psychological concepts of social relations (such as social support). Thus, through a partly abstracter operationalisation, social capital could be seen to tap into a wider range of social processes, beyond those between people and groups.

Regarding its relation to health, social capital is correlated with a wide variety of positive health outcomes. Health is promoted by social capital both at the individual and the collective level through a large number of processes (see [34] for an overview). However, being a broad and abstract concept, the social capital literature tends to tell us less about its exact underlying mechanisms. Most literature we came across relies on theory that has its roots elsewhere. For example, we find traces of social capital and reference to the concept in the work of Jetten and colleagues [35], a literature that is, as mentioned before, strongly related to cognitive social capital. And in the literature on disaster and community resilience, social capital is sometimes considered to be a key condition for resilience; but the workings of social capital are accounted for by other concepts and processes, such as social support and network structures [36]. When it comes to drawing conclusions, we find that many studies then move up to the abstract level and claim “social capital is associated with…”. Or, to put it simply, social capital has large theoretical overlap with other concepts when broken down, but is then often presented as a distinctive concept.

### Present research

The different concepts of social relations, *Social Support*, *Multiple Group Membership* and *Social Identification*, and *Social Capital* as discussed above, promote health and wellbeing through various processes. They are ostensibly distinct, emerging from separate literatures and having (mostly) distinct theoretical origins. However, we also note that there is a considerate amount of theoretical overlap. For example, the concepts of *social capital* and *multiple group membership*, share the assumption that social relations are ‘cumulative’, in a sense that the more (relevant) ‘groups’ or ‘social nodes’ one has, the better. Additionally, as we outline above, the theoretical conceptualisation of social capital contains elements of support and identification. In a somewhat different comparison, we recognise that the concepts of *social identification* and *social support* both operate through the process of ‘support’. Specifically, a shared social identity has shown to give one access to social support and so we could argue that both concepts seem to work through the same process.

Based on our evaluation of the different literatures and approaches, there appears to be scope for integration. This paper will adopt an empirical approach to examining the overlap and/or distinctiveness of, in first instance, social support, social identity and multiple group membership (Study 1). Following our initial evaluation in Study 1, we will also include the more overarching concept of social capital in Study 2 to examine how it relates empirically to the afore mentioned concepts of social relations. We hypothesise that, based on the discussed literature, these constructs should be identified as separate entities in our analyses. We will test this by performing several confirmatory factor analyses (CFA), testing a factor structure in line with the theoretical constructs (e.g. a factor for identification and a factor for social support). However, it is very well possible that all these constructs have a “general factor” in common, for example due to sociability or due to having social relations in the first place. It is best possible to appreciate the unique value of distinct components such as support or identification, after taking that general factor into account. An exploratory bi-factor approach is suited to investigate this issue.

## Study 1

### Method

#### Participants and procedure

All procedures performed in studies involving human participants were in accordance with the ethical standards of, and approved by the ethical board of the department of psychology of the University of Groningen, The Netherlands (research code for these specific studies ppo-015-085). All participants gave informed consent in writing or by confirming their consent in the first online questionnaire. Participants were members of a representative panel of inhabitants of the province of Groningen in the Netherlands. In this paper we analyse the data from two time points (study 1a and b), at which a battery of questions about social relations were included (these measures were not included at the other time points). The dedicated panel consisted of four thousand, five hundred seventy-seven inhabitants of the province of Groningen, The Netherlands (1977 males; *M*_age_ = 56.54 years, *SD* = 14.78, range = 16–93). During two years these participants were asked to fill out five questionnaires. The study was run among a selection of participants both within and outside a region affected by induced earthquakes. The study was financed by the National Coordinator Groningen to assess the impact of gas extraction on social relations, safety perceptions and health. To achieve a representative sample, also for rural areas, less densely populated areas were oversampled. The majority of questionnaires were filled out on-line and a small number were completed by post (*n* = 154), within four weeks of the invitation.

Study 1a (*N* = 3934, 1967 males, *M*_age_ = 56.54 years, *SD* = 14.79, range = 16–93) included measures of identification and social support. Study 1b (*N* = 3153, 1547 males, *M*_age_ = 57.74 years, *SD* = 14.19, range = 16–93) was conducted at a different moment in time and included measures of identification and multiple group membership.

#### Measures

*Identification* was measured in relation to two groups: neighbours versus family/friends in Study 1a. The first target of identification was the *Neighbourhood/village*, and was measured with a single item measure: “I feel connected to the people in my neighbourhood” ([37]; see also [38] for a comparable single item measure). The second target of Identification *Identification with family/friends* (in Dutch: “naasten”). This was operationalised in an identical manner as identification with neighbourhood but targeted at family/friends and adapted slightly to fit this target (so “I feel connected to my family/friends”). Study 1a also included measures of *social support* from two targets. *Social support from the neighbourhood* was measured by a single item “I receive the help and support I need, from the people in my neighbourhood or village” (adapted from [8]). The second target, *Social support from family/friends* was measured with the same item but adapted to target family/friends: “I receive the help and support I need, from my family/friends”.

*Identification* in Study 1b was measured with a four-item scale [5, 39] (e.g., “I feel committed to the people in my village or neighbourhood”; 1 = “I totally disagree”, 5 = “I totally agree”, Cronbach’s α = 0.89). Like in Study 1a, *identification* was also measured for two targets in Study 1b, differentiating between village or neighbourhood versus family/friends (Cronbach’s α = 0.91).

*Multiple Group Membership* was measured in Study 1b with a four-item scale (Cronbach’s α = 0.93) capturing the number of groups somebody belongs to [5] (e.g., “I have strong ties with lots of different groups”; 1 = “I totally disagree”, 5 = “I totally agree”). To be clear, we did not measure the actual number of groups, but the *perception* of the number of groups. All measures used in Study 1 are presented in Table 1.

### Results

#### Study 1a

A preliminary correlational analysis was performed to explore the relations between the four items measured (see Table 2). Interestingly, the identification items (neighbourhood & family/friends) are highly correlated with the respective support items (neighbourhood & family/friends; *r* = .67 and *r* = .76). Contrary to expectations that the items might differentiate between the constructs of identification and support, the results suggest that items referring to particular targets tend to correlate very highly. Conversely, the correlations between the two support items and the two identification items are very low (*r* = .32 and *r* = .27). Because there are only four items and because the correlations speak for themselves, carrying out a factor analysis does not make much sense. It is clear that items cluster not by concept, but by target group.

**Table 2:** Means, standard deviations, and correlations of four variables on social support from neighbourhood, identification with neighbourhood, social support from family/friends, identification with family/friends.

	*M*	*SD*	1	2	3
1. Support Neighbourhood	3.97	1.08			
2. Identification Neighbourhood	3.95	1.10	.67**		
3. Support Family/Friends	4.54	0.86	.32**	.26**	
4. Identification Family/Friends	4.67	0.74	.26**	.27**	.76**

*Note*. *M* and *SD* are used to represent mean and standard deviation, respectively.

* indicates *p* < .05.

** indicates *p* < .01.

#### Study 1b

In a later measurement, we had the opportunity to include a much broader range of identification measures. Unfortunately, the limited space in this wave meant we were not able to include any of the social support items. We did however find it meaningful to validate the patterns we found in Study 1a in more detail. Accordingly, we could examine the factor structure of the broader range of identification measures, at a different point in time. A preliminary correlational analysis was preformed to explore the relations between variables. These correlations are presented in Appendix A in S1 Appendix. The pattern of correlations is quite similar to results from Study 1a, differentiating three groups of variables that correlate highly, based on the target groups that the items refer to. We performed a confirmatory factor analysis to evaluate the factor structure of the data based on the underlying theoretical concepts: one factor for multiple group membership and one factor for the identification measures. Results revealed a model with a bad fit: CFI = .64, RMSEA = .22, SRMR = .12, according to cutoff criteria provided by Hu and Bentler [40]. A closer examination of the factor loadings suggests a different structure. Factor loadings for all items in the *Multiple group membership* factor were high, ranging from .71 to .86. The *Identification* factor loadings ranged considerably more, ranging from .48 to .82. A distinction here can be made, between *identification with neighbourhood* items forming the lowest four factor loadings (.48 to .58), and *identification with family/friends* (.73 to .82). We also note that the correlation between the two factors is .45, indicating that a general factor might account for a portion of variance.

We next tested a model in which we specified three factors, based on their target and not on their underlying theoretical concepts: one for the *Multiple Group Membership* items (which we consider to be target unspecific, based on its operationalisation), another for *Neighbourhood Identification* items and the third for *Identification with family/friends* items. Results revealed a slightly better model fit: CFI = .86, RMSEA = .15, SRMR = .06. Though the model fit has improved this does not yet represent a good fit, according to the criteria [40].

As a CFA did not yield a satisfactory fit to the data, a more exploratory approach was taken. This allowed for different alternative structures of the data, other than the factor structure based on theoretical concepts. To further analyse the relationship between the items on *Multiple Group Membership*, *identification with Neighbourhood* and *Identification with family/friends*, an exploratory bi-factor analysis was conducted with the function “omega” in the Psych package in R [41], which is an exploratory factor analysis originally developed by Schmid and Leiman [42]. A bi-factor analysis takes into account a general factor and gives us the opportunity to distinguish the unique variance explained by the target factors from the common variance explained by the general factor (the overarching concept). For a more detailed demonstration of the use of bi-factor modes see [43]. In this bi-factor model the general and the group factors are assumed to be orthogonal, in order to enable us to estimate the unique variance accounted for by any components [44]. The fit of the bi-factor model is an improvement compared to previous models. Results revealed a substantially better fit, albeit not perfect: CFI = .92, RMSEA = .11, SRMR = .15. Adding a general factor to the model appears to explain considerable extra variance. Indeed, the common variance explained by the general factor is quite high: 67%. This suggest that of all the common variance, more than half is attributable to the general factor. In other words, even though identification with various target groups and multiple group memberships are quite distinct concepts, the items measuring these various concepts of social relations also have a common core of what might be called "groupiness".

We report the remaining reliability results from the bi-factor analyses using the Omega coefficient [45]. Omega is a measure of reliability that can be inferred from the bi-factor analysis and tells us the proportion of total score variance that can be attributed to all items, to each factor and to the general factor [43].

**Fig 1:**
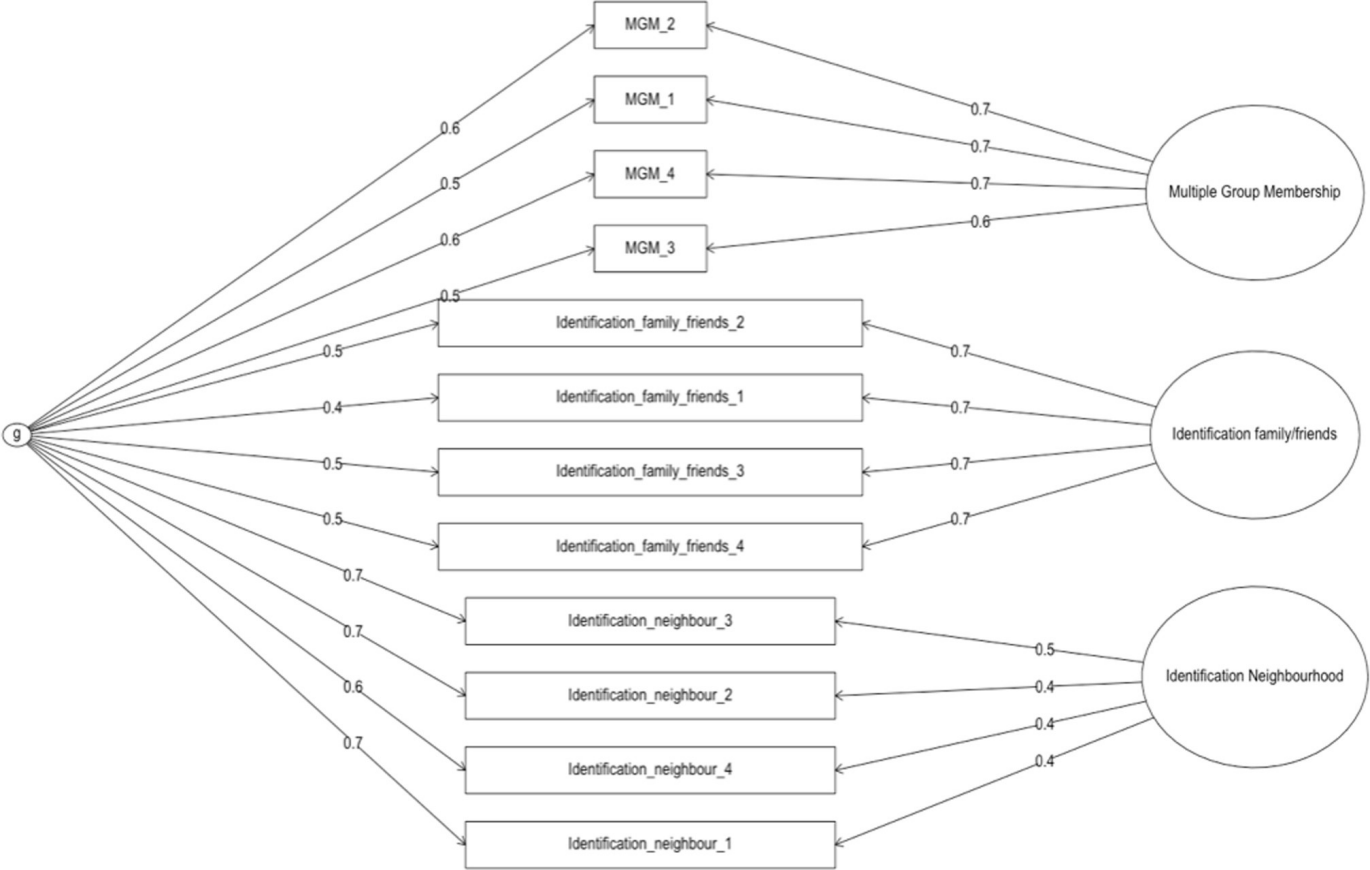
Bi-factor structure of measures in Study 1b. Item names correspond to measures listed in Table 1 respectively. Numbers on arrows represent factor loadings.

The bi-factor analysis shows that in addition to the overarching variance that is shared among all the items referring to groups and group membership, a distinction can be made between three dimensions predicting *Multiple Group Membership* (F1), *Identification with family/friends* (F2) and Identification with *Neighbourhood* (F3). Fig 1 shows the results of the bi-factor analysis. This analysis confirms the impression from the confirmatory factor analyses: three different dimensions can be identified, over and above a general factor. What is clear from the bi-factor analysis is that items are grouped based on the target and not on the general concept (e.g. identifying with a relevant group). What is also clear is that the factor loadings of the general factor tend to be acceptable, and those of the components as well.

Reliability of the general factor is measured by “Omega H” and tells us the proportion of total score variance that can be attributed to the general factor, taking the factor structure into consideration. Results revealed an Omega H of .67. When we compare this to the Omega of the total scale, this means that 67% of total score variance is attributable to the single general factor. A sizable part (28%) of the variance in total scores is attributable to the multidimensionality through factors.

Reliability of the sub scales (individual factors) is indicated by “Omega S’ and can be interpreted in identical way as it is for the other Omega parameters: Omega S is the percentage of subscale score variance uniquely attributable to the factor after deducting the variance due to the general factor. The results revealed the following: the Omega S values for *Multiple Group Membership* (0.56), identification with *Family/Friends* (0.62) and identification with *Neighbourhood* (0.27). It tells us that it is generally meaningful to make a distinction between these factors as their reliability estimates increase, after controlling for the variance caused by the general factor.

## Discussion

The results from studies 1a and 1b suggested a different factor structure in the data than would be expected based on the literature: the literature suggested measures to group according to their concepts, but results revealed them grouping according to “target”. Moreover, we find strong empirical overlap between the different concepts: 67% of total score variance is attributable to a single general factor. This means that with regard to distinguishing social support, multiple group membership and social identification, we have initial evidence that these concepts also can be operationalised quite well as one general factor. However, analyses in studies 1a and 1b were fairly limited both in number of items included and number of concepts measured in a single study. Importantly, we also felt that the inclusion of a more overarching measure of social relations, social capital, could serve to fill the caveat in our current empirical test of social relations. *Social capital* is in our eyes the most extensive concept, capturing a large set of characteristics of social relations. *Social capital* encompasses elements of all three concepts measured previously, such as perceptions of connectedness and social support. Overall it is defined as referring to resources (support, information etc.) that are available within one’s social network [46], and features both a structural component (what we do and receive) and a cognitive component (what we feel) [47]. The questions we sought to answer in Study 2 were whether we could replicate results of Study 1, including all three previous factors (social support, social identification, multiple group membership). Moreover, the addition of social capital meant we could assess whether social capital might better empirically capture the concept of social relations, in itself. The same (factor) analyses used in Study 1b will be conducted in order to describe the factor structure of the data in Study 2.

## Study 2

### Method

#### Participants

All procedures performed in studies involving human participants were in accordance with the ethical standards of, and approved by the ethical board of the department of psychology of the University of Groningen, The Netherlands (research code for these specific studies ppo-015-085). All participants gave informed consent in writing or by confirming their consent in the first online questionnaire. The current study included 2912 participants (42.51% male, *M*_age_ = 53.94 years, *SD* = 10.75, range = 23–84) of the “Lifelines” panel. Lifelines is a multi-disciplinary prospective population-based cohort study examining through a unique three-generation design the health and health related behaviours of 167729 people living in the North of the Netherlands. It employs a broad range of investigative procedures in assessing the biomedical, socio-demographic, behavioural, physical and psychological factors which contribute to the health and disease of the general population with a special focus on multi-morbidity and complex genetics. The study was run among a selection of participants both within and outside the earthquake region and was presented as research on social contacts, feelings of security and health. Participants first answered questions about social relations before filling in any questions in relation to gas extraction. As in study 1, the present study was also part of a larger study. Financing of this study occurred via a grant by the National Coordinator Groningen to assess the impact of gas extraction on social relations and health.

#### Measures

An overview of additional scales and items used in Study 2 is presented in Table 3. *Identification* with *neighbourhood/village* was measured in two ways, a single item as measured in Study 1a and a 4-item scale as was measured in Study 1b. *Identification* with *family/friends* (in Dutch: “naasten”) was measured merely by a single item, in the way it was in Study 1a.

**Table 3:** Additional measures used in Study 2.

Scale name	Scale Items
Social support	1. My family really tries to help me
	2. I get the emotional help and support I need from my family
	3. I can talk about my problems with my family
	4. My family is willing to help me make decisions
	1. My friends really try to help me
	2. I can count on my friends when things go wrong
	3. I have friends with whom I can share my joys and sorrows
	4. I can talk about my problems with my friends
	1. There is a special person who is around when I am in need
	2. There is a special person with whom I can share my joys and sorrows
	3. I have a special person who is a real source of comfort to me
	4. There is a special person in my life who cares about my feelings
Adapted Social Capital Assessment Tool^b^^,^ ^c^	Group membership items
	1. In the last 12 months have you been an active member of any of the following types of groups in your community? - Village or neighbourhood association - Religious group - Sports group - Work related/trade union - Political group - Protest group
	Support from groups item
	2. In the last 12 months, did you receive from the group any emotional help, economic help or assistance in helping you know or do things?
	Support from individuals items
	3. In the last 12 months, have you received any help or support from any of the following people? - Family - Neighbours - Friends, who are not neighbours - Politicians - Government officials/civil service - Someone from a charitable organisation or voluntary organisations - Religious leaders/workers
	Citizenship activities items
	4. In the last 12 months, have you joined together with other community members to address a problem or common issue? 5. In the last 12 months, have you talked with a local authority or governmental organisation about problems in this community?
	Cognitive social capital items
	6. In general, can the majority of people in this community be trusted? 7. Do the majority of people in this community generally get along with each other? 8. Do you feel as though you are really a part of this community? 9. Do you think that the majority of people in this community would try to take advantage of you if they got the chance?

^a^Items were measured on a 5-point Likert-type scale from *strongly disagree* (1) to *strongly agree* 5)

^b^Items were measured by *no* (1) or *yes* (2)

^c^Items were summed to create Social Capital variable: S_Capital.

We measured *Social support from the neighbourhood* with a single item: “I receive the help and support I need, from the people in my neighbourhood or village” (adapted from [8]). *Social support from family/friends* was measured with the same item but adapted to target family/friends. In addition, a set of eight items was used to measure *social support from friends or family* [8]. The latter eight items make a specific distinction between *friends* and *family*, e.g.: “My family really tries to help me” versus “My friends really try to help me”. Four items concerning the social support received from a “*Special Person”* were used from the same scale [8]. These four items all depict a specific kind of support from a special person (e.g., “There is a special person in my life who cares about my feelings”). In sum, two items concerning the support from either the neighbourhood or friends/family were included and a twelve-item scale was included to measure support from either friends, family or a special person.

*Multiple Group Membership* was measured with the same four-item scale used in study 1b.

*Social capital* was measured by nine different items adapted from the Adapted Social Capital Assessment Tool (A-SCAT; [48]). The A-SCAT is comprised of five categories of items referring to: *Group membership*, *Support from groups*, *Support from individuals*, *Citizenship activity* and *Cognitive social capital*. All items were scored (dichotomously) as in the original A-SCAT, except for the *Support from group items*, that was framed as a follow up question from the previous question (“In the last 12 months, did you receive from the group any emotional help, economic help or assistance in helping you know or do things?”). This was done to shorten the length of the questionnaire. Also, the wording of some items was adapted to fit the context of the study. Tetrachoric correlations were conducted to test the internal reliability of the social capital scale (Appendix D in S1 Appendix), showing very low correlations between items and showing no clear pattern of clustering items. We also performed a series of correlational analyses including the measure of social capital as single items in relation to the other concepts (Appendix C in S1 Appendix). That is, each dichotomous item was entered into the model with our other items that measure social relations. The results showed that the majority of social capital items did not correlate at all with any of the items from other concepts, with a correlation coefficient ranging from .01 to .28. It was therefore decided to include social capital as a ‘sum’ of all the items, as that would be the only way to meaningfully include it further in our analyses. Indeed, it makes little sense to do a factor analysis with items that do not correlate. Appendix B in S1 Appendix shows the correlation matrix with social capital as a sum and shows that this yielded higher correlations, justifying the use of a single sum score (Cronbach’s α = .96). All social capital items were summed to a total score, a higher score indicating more social capital.

### Results

A correlational analysis was performed to explore the initial relations between variables. These correlations appear to show high correlations between items that refer to the same targets (neighbourhood and family/friends). A series of factor analyses was conducted to test this impression.

We first conducted a confirmatory factor analysis in which we defined factors in line with theoretical conceptualisations (Social Identification, Multiple Group Membership and Social Support). Social Capital was not included in this confirmatory factor analysis, due to the fact that we computed social capital as a single variable. Treating this single variable as a separate factor would not result in a meaningful analysis. This model showed a bad fit (CFI = .64, RMSEA = .13, SRMR = .12). Correlations between the three factors ranged from .36 to .45. The results suggest that the assumed factor structure is not a good description of the covariance among items. The majority of the items have high factor loadings. However, some items concerning specific targets tend to have much lower loadings (e.g. the identification item targeting family/friends has a factor loading of .34, whereas all other items in that factor range between .71 and .87).

From Study 1b and the inspection of correlations in study 2, we can infer that one reason for the poor fit may be that items share a lot of common variance. We therefore included a general factor in the next model. Results revealed that the fit for the model improved (CFI = .78, RMSEA = .11, SRMR = .11). Though the fit of the model improved by adding a general factor to the equation, the latter indices by no means represent a good fit. The factor loadings on the general factor range from .18 to .87. This means that some items are highly connected to the general factor, but that others are very loosely connected. Moreover, within ‘scales’ (e.g. social support), item factor loadings onto the general factor vary greatly.

**Fig 2:**
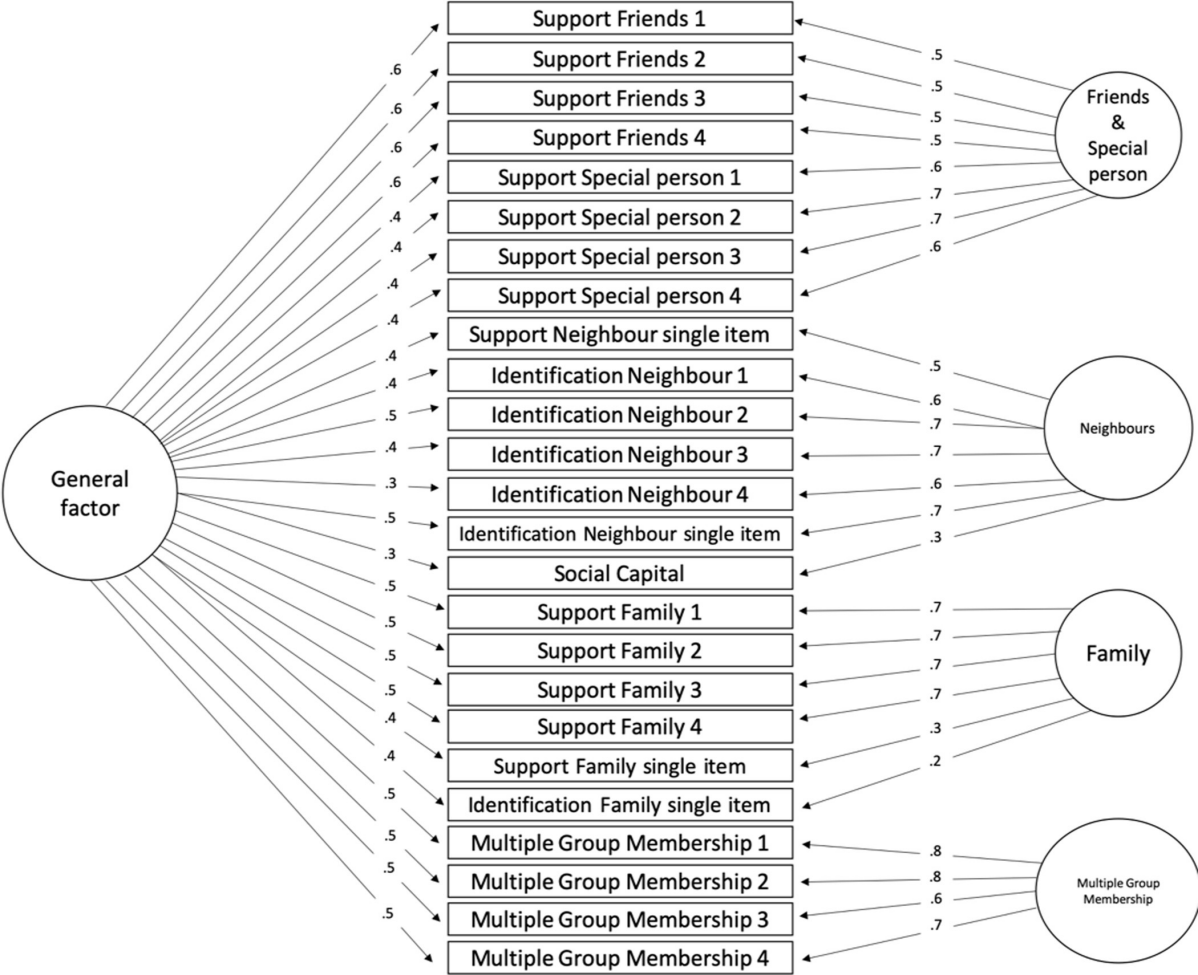
Bi-factor, Omega results with 4 factors. Item names correspond to measures listed in Tables 1 & 2 respectively. Numbers on arrows represent factor loadings.

As in study 1b, the final step in our analyses was to perform an exploratory bi-factor analysis with the function “omega” in the Psych package in R [41]. In this analysis we also included the single measure of social capital, because we wanted to see how this concept factors in with other multidimensional measures. The four-factor model, visualised in Fig 2, distinguishes *friends* (F1) from *family* (F3). Moreover, the factors in the latter model appear to be even clearer, loading items onto factors that are concerned with particular target groups or persons, rather than with support or with identification. Results revealed a much better model fit: CFI = .89, RMSEA = .07, SRMR = .11. As explained in study 1 however, reliability indices are more indicative when reporting the results of a bi-factor analysis.

As discussed in study 1 we use the Omega coefficients to determine the reliability of a model. Omega H, measuring the reliability of the general factor, was .63. This means that 63% of the total score variance is attributable to the general factor, leaving 33% of total score variance due to the factors. When studying the reliability indices of the subscales (the four factors), we find the following values for Omega S: *Friend/special person (F1)* = .49, *Neighbourhood (F2)* = .59, *Family (F3)* = .46, *Multiple Group Membership (F4)* = .63. These Omega S values tell us that all four dimensions are reliable and account for a reasonable amount of unique variance in the total scores, even when controlling for the influence of the general factor. Again, as was the case in study 1, these results show a meaningful distinction between the targets of social relations and not between concepts. Items relating to a “special person” turn out to be clustered together with items relating to support from friends. Where the three-factor solution already showed us that a “special person” is related to friends/family and not the neighbourhood, the four-factor model specifies this further by grouping a “special person” specifically with the friend items. *Multiple group membership* items, finally, form a single factor (F4) in the 4-factor model. Social capital, interestingly, does not seem to relate clearly to any of the factors. It is somewhat related to the general factor and somewhat to neighbourhood relations (and not, to our surprise, to multiple group memberships), specifically none of the social capital factor loadings yielded above .3. An exploratory five factor model, from which one might expect a separate factor for “special person” or for *social capital* (not reported in detail here) does not explain substantially more variance, we should mention.

### Discussion

Building on Study 1, the current study also included a measure of social capital. Unfortunately, the internal reliability of this established measure of social capital was high only because of the very large number of items [49] and we had to resort to including only a one item sum score in our analyses. Perhaps not surprisingly, this sum score also did not strongly relate to any of the other factors in the analyses, the strongest relation being with the general factor and that of neighbours. As we will outline further in the General Discussion, the fact that we had to operationalise social capital in this manner, makes it somewhat problematic to draw conclusions with regard to social capital. The fact that social capital scores were somewhat related to the general factor and neighbours factor means we cannot draw strong conclusions, but does give us some pointers to an empirically based interpretation of what this scale taps into. Particularly the connection with neighbours, over and above the general factor, suggests that *social capital* measures of this kind appear to be not only associated with a general sense of social relatedness but are also slightly tilted to the unique aspects of one’s relations to the immediate non-kin community: perhaps the focus in these measures is less on the warmth of those relations and more on the *availability* of those community relations.

Yet, importantly, Study 2 replicates findings from Study 1: measures of social relations group according to “target” and not according to their theoretical conceptualisations. The fact that we were able to include an extensive measure of social support that included several different targets (e.g. also special person, friend) and yet revealed the same pattern of results, makes this study even stronger.

We discuss broader implications of the two studies taken together in the General Discussion.

## General discussion

Social relationships are fundamental to human life: people rely on those relations because close others provide them with support, because their extended social networks give them access to others who have information, resources and valuable connections, and because the different social groups they belong to provide them with meaning, a sense of belonging and identity (and sometimes a social stigma in relation to other groups in society). The nature of these different types of social relationships has been conceptualised in different ways across different research traditions in clinical psychology, social psychology and sociology. Each of these literatures has a different perspective on what types of relations might be beneficial to us, and why. But importantly, they all converge on the same conclusion: social relations are fundamental for human wellbeing and—more specifically—for health outcomes. Indeed, there is substantial evidence that each of these concepts are predictive of important health outcomes (e.g. [1, 17]). Moreover, numerous other research fields have pointed out the importance of social relations. For instance, in political psychology, social relations have proven to be a valid predictor of political engagement [50]. Some scholars have even argued that a rich amount of social relations can lead to more economic development and community wellbeing [2, 29, 51].

A question that has received relatively little empirical attention thus far is whether these different measures of social relationships are conceptually distinct or whether there is scope for integration across these traditions because of the presence of one underlying concept. The present work aimed to answer this question. Specifically, we studied the relations between concepts of *Social Support*, *Multiple Group Membership and Social Identification* across two studies in which we assessed different factor structures in relation to an overarching measure of social relations. In addition we also attempted to incorporate *Social Capital* in our investigation, in Study 2. Results provided evidence for a strong general factor, explaining a large amount of variance. There is also support for separate dimensions that explain modest amounts of variance. Importantly, from a theoretical perspective, is the finding that these dimensions do not cluster around a conceptual dimension, but rather cluster on distinct targets of social relations such as neighbours or family members. We return to this later.

### Strong general factor

Importantly, the present work demonstrates that there is a large amount of empirical overlap between Social Support, Social Identification and Multiple Group Membership. Indeed, both empirical studies provide evidence for a strong general factor, meaning that there is a considerable amount of variance explained by all measures taken together: The proportion of variance in total scores that can be attributed to the general factor is 67% in Study 1 and 63% in Study 2 (cf. [52]). What this means is that measures of Social Support, Social Identification and Multiple Group Membership and, albeit to a lesser degree, *social capital* all share considerable common variance, presumably because people’s understanding of the very different aspects of social relations that these measures are supposed to tap into, share (at least in the perceptions and responses of individual respondents) a common core feature. The central question is what this common core should be understood to be. Looking at the factor scores does not reveal a single item that loads extremely highly on the general factor. The item least strongly predicted by the general factor is social capital, although the range of factor loadings is not huge. Across the board, all items load approximately equally strong: thus, we are really looking for a common element underlying all of the items. In order to answer the question what this common factor is, we should first examine the full pattern of results and the individual components.

### Distinguishing between who we relate to

We hypothesised that our data could be structured according to the theoretical constructs of social support, multiple group membership and social identity. Consequently, we conducted a confirmatory analysis based on those theoretical assumptions. The results were not convincing however: factor analyses based on these theoretical constructs, yielded models with a bad fit across all studies. Following those results, we conducted exploratory bi-factor analyses. This analytic method allows one to identify specific components in the presence of a superordinate dimension (or general factor). This resulted in a model with an improved fit, and one result was that we demonstrated the presence of the afore mentioned general factor. But across the studies there are also individual components that add sufficient unique variance to take note of. The present work reveals that these components cluster based on the target of the social relationships that is mentioned in the individual items. That is, conceptualisations that specify a target in their operationalisation, cluster around those targets together with other concepts. However, concepts that do not specify a target would most likely be associated with a general factor, but not with those target factors. For example, the measure of multiple group membership does not specify *who* the groups we belong to contain and is clearly also identified as a separate factor. We think this is the result of the slightly different conceptualisation that multiple group membership is, namely that of the quantity of the relation. We will return to this later.

Across studies we find a consistent pattern that items cluster by targets and target groups such as one’s *neighbourhood*, *family* or *friends*. When partitioning out the variance of the general factor, we find that constructs relating to *friends/special person*, *neighbours and family* explain 49%, 59%, and 46% of unique variance respectively [52]. This implies that we have considerable common variance, but also the unique variance is near the threshold of .5 at which a component scale has sufficient reliability [43]. An even higher percentage was observed for the construct of *multiple group membership*, which will be discussed later. We see a similar pattern across both studies: the subscales that refer to specific targets tend to have adequate reliability. Thus, over and above the general factor, it makes *empirical* sense to differentiate subscales based on *who* we relate to, more so than on how we relate to one another (e.g., support, identification etc.).

The necessity to distinguish among different targets has important theoretical implications, when studying social relations and its broad array of associations. For instance, a large body of literature has pointed to the health benefits of identifying with social groups [27] or the benefits of receiving support *in general* [8], without offering a clear rationale for which groups matter, or without disentangling who is giving support. Our results show that it is possible to meaningfully differentiate among social groups. This opens the possibility of systematically examining whether it is just any relevant social group that can be beneficial to one’s life, or whether specific groups are more beneficial, perhaps depending on social context. This approach would be consistent with a recent meta-analysis suggesting that the strength of the relationship between group identification and (lower) levels of depression varies depending on group type (e.g. being lower in small groups; [53]). This approach is also consistent with research on *social support* highlighting the specific characteristics of support offered by family members [8, 54]. To be clear, we are not suggesting that the literature has entirely overlooked the relevance of specific target groups such as family or neighbourhoods. Rather, the point we are making is threefold. One is that we have not been able to identify any studies that distinguish between the effects of targets within a single study—there does not seem to be an empirical literature that examines these between-target differences. The second is that several measures exist which integrate relations across different target groups together into a single measure of social relations (see the afore mentioned study by [8])—the risk here is that between-target differences are obscured. The third point is that even though prior research shows that humans perceive target groups very differently (e.g., [55]), we are not aware of any literature that has theorised the grounds upon which these targets might have different effects for outcomes such as health and individual well-being.

The good news is that the present work suggests that there is a sound empirical basis upon which future empirical work can meaningfully distinguish such social relations from each other, based on who they target, in order to enhance our understanding of whether certain social relations are more important for a variety of outcomes, than others.

The results also show that the targets of the relationship (e.g. neighbours, friends, sports group etc.) are more important than the type of relationship (support, identification) one has with an individual or group. This finding that items referring to support and identification load on the same factor suggests that there is scope for further theoretical integration of these distinct literatures on support, identity and perhaps even social capital. It also means that future empirical research that aims to differentiate between the three needs to operationalise variables in a different way in order to achieve its objective. For example, Swartzman, Sani and Munro [56] investigated the relationship of *social support* (measured with the “MOS social support survey” by Sherbourne and Stewart [57]) and *family identification* (measured with the “Group identification measure” by Sani et al. [58]) with health. A sample item of the former is "how often is someone available to take you to the doctor if you need to go". A sample item of the latter is "I have a sense of belonging to my family." From our results, we would recommend that future research prevent that the distinction between theoretical concepts (support vs. identification) and the distinction between social target groups of interest (a special person vs. neighbourhood) is being confounded (as we have seen in some past research). Otherwise one cannot tell whether any effects are due to the concept of interest or the target that items refer to. More generally, judging by the fact that we could more easily differentiate between components based on targets, we suggest that the more fruitful avenue for future research is to develop a better and more theoretically grounded understanding of which social relations are the most likely to benefit health and wellbeing. We speculate that the interaction between the aspect of health (e.g., mental or physical) and the context in which health is being threatened (e.g., whether it is a private health issue, a public one or a collective threat) may play some role in explaining the relevance of certain groups such as family or neighbours.

### Quality versus the quantity of relationships

Although we found strong evidence for overlap in the concepts of *social support*, *social identification*, *multiple group membership* and to a lesser extend *social capital*, we also found that *multiple group membership* can be clearly distinguished as a separate dimension. After partitioning out the common variance associated with the general factor, the unique variance that the dimension of *multiple group membership* accounted for was 56% (Study 1) and 63% (Study 2), respectively (cf. [52], p. 141). Although *multiple group membership* is rooted in the social identity approach, the literature also provides a clear distinction between identification measures and a multiple group membership measure [5]. *Multiple group membership* is based on the idea of a more complex and varied self-concept. The idea of a complex self-concept was consequently conceptualised as the possession of multiple identities [3, 27]. The affiliation with multiple groups in turn, has shown to be beneficial in buffering against for example depression [3]. Simply put, *multiple group membership* refers to the perceived *number* of significant groups one is affiliated with (quantity), whereas the group *identification* measure focuses on the degree of identification (quality) with one particular target group.

The theoretical implication of this result is that we should draw a clear distinction between the quality of relations with target groups and the quantity of target groups available to an individual. In other words, our research suggests that it is both possible and therefore potentially fruitful to study the impact of the number of relationships a person has access to, in addition to examining their quality (e.g. in terms of identification and support provided within them). Here we notice a clear parallel to a debate in the *social support* literature (e.g., [59, 60]), regarding whether *perceived quality of support* or *actual support network* is more beneficial for health. We know at least from the literature that both received and perceived social support each can have an important function for health: more received social support can lead to more perceived social support in the long run. Perceived social support is in turn associated with an improved mental health [61].The distinction between the quality and the quantity of social relations, is also discussed in the literature on social isolation and loneliness (e.g. [62]). In sum, the current results underscore that future research would do well to consider not just the quality but also the quantity of social relations: each may have a differential impact on, or affect health and wellbeing for different reasons.

### Interpreting the general factor

So far, we have concluded from our results that meaningful distinctions can be made on the basis of targets of social relations and on the basis of their quality vs. quantity. But as we noted at the start of the discussion, the ability to differentiate among distinct components should not ignore the presence of a strong general factor. Our central finding, we suggest, is that all of these components of social relations have a lot of empirical overlap. This naturally raises the question: what is this commonality among these measures? We answer this question in two ways, first by exploring the theoretical overlap among the approaches considered in this paper, then by considering how future research can help us address the issue.

The different concepts included in this research come from distinct literatures which each have their own distinct theoretical bases. *Social support* refers to the beneficial psychological and material resources provided by one’s social network. *Social support* could be defined as something that is exchanged or given. Coming from a different perspective on social relations, the *Social Identity Approach* (SIA) specifically highlights the importance of group membership for the individual’s sense of self [5, 22]. Or to put it simply, social relations revolve around someone’s understanding of who they are in relation to the groups they are members of. Social relations, according to SIA, are therefore more than just connections between individuals within a network. SIA, in contrast to *social support*, also does not presuppose that any physical exchange takes place or even that physical co-presence is required. As set out previously, *multiple group membership* builds on the theoretical framework of SIA, but distinguishes itself by emphasising the *quantity* of the relationship (again, a mental representation of the many groups one feels belongingness to). Lastly, the concept of *social capital* is in our eyes the most extensive concept, capturing a large set of characteristics of social relations. *Social capital* refers to resources (support, information etc.) that are available within one’s social network [46], and features both a structural component (what we do and receive) and a cognitive component (what we feel) [47].

The question is, what do these different concepts and processes have in common that can explain a strong general factor? Our suggestion is that all revolve around what one can refer to as *the perceived presence of others in one’s life*. Thus, cutting through the theoretically driven concerns with groups vs. individuals, the concerns whether others offer concrete support or other benefits such as access to scarce resources, whether they provide a sense of identity, belonging, place and/or meaning, may be quite simply an overriding sense that there are other people in one’s life that one can relate to, share things with and bond with. The theoretical implication is that across these approaches, there is scope for integration. Thus, in addition to theories about why others matter in terms of support, other resources or social networks, or why they matter for the psychological benefits that group membership may bring, we may also need to further elaborate a coherent perspective on what this sense of presence of others encompasses: what does it mean to have a sense of presence of others?

Theoretically *social capital* seems best equipped to bring together elements across the broad spectrum of what this feeling entails. It has served as an overarching “umbrella” term across research fields in the past (e.g. [36]), making it a useful concept yet also with a major limitation: it compromises many different facets of social relations into a single scale. Social capital has the scope to encapsulate both social support and social identification (and does so in a way, in its operationalisation). It is therefore quite ironic and surprising that the measure, or specifically the operationalisation thereof, of social capital we included in our research turned out to be the *least strongly correlated* with other measures of social relations. It would be premature to conclude from this that social capital can be ruled out as an important overarching concept, however. We believe there is reason to develop and investigate this measure further. The measure we used to operationalise social capital [48] may be seen as an attempt to integrate items relating to support as well as ‘connectedness’, but the methodology does not ask about the extent to which one feels connected: all items are dichotomous, referring to whether one has certain elements or not, that are understood to be part of social capital. And though we acknowledge, that the social capital measure includes items referring to *feeling connected*, through the dichotomous operationalisation this specification seems to be lost. Especially, when one uses a sum of the items as we have done. Therefore, the reason that our measure of *social capital* does not correlate well with any of the other approaches may be that it only captures the *being* connected, whereas the other measures are more about *feeling* connected. And as the literature on loneliness shows, the correlation between feeling connected and the actual number of connections one has tends to be low [63].

A strong general factor, representing what we have called ‘the perceived presence of others in one’s life’ (or more succinctly, feeling connected) may be an interesting and strong finding across these studies, but it also creates a theoretical and practical challenge for future research. How should future research on the relationship between social relations and health, in each of the three approaches covered in this paper (support, social identity and social capital), make use of this finding? Our findings clearly challenge the utility of studying separate mechanisms underlying the relationship between social relations and desirable outcomes (e.g., based on either social support or levels of identification with a group) in isolation of one another. The core problem is that measuring a single concept cannot meaningfully distinguish between different factors that could be at work when we find any effects on for example health and wellbeing: finding that support is beneficial, or identification, can potentially all be explained by the general factor we have identified—that is health benefits across these three theoretical approaches may simply be due to feeling connected. From the literature on social relations’ effect on health and mortality one could argue a similar conclusion can be drawn from the results: the strongest predictor of these outcomes is not a single variable but a complex mix of different aspects that together make up our "social life". Our recommendation is that future research should study the impact of social relations, by focusing on more than just social identification, support or capital as single predictor: we should use questionnaires to tap into the general factor and (where necessary and where suitable power to detect effects is available) distinguish among distinct components related to targets of interest. Using such a methodology allows one to tease apart the predictive utility of both the general factor and the unique components. This road offers more than just methodological refinement: it raises new theoretical avenues and scope for integrating and refining what are, oftentimes, distinct and separate literatures.

### Limitations

Some limitations also need to be addressed. Our studies only include ‘proximate’ targets such as neighbours, friends and family. This means that our research ignores targets such as social categories. Furthermore, we acknowledge that some of the measures, that of social capital in particular, have been adapted to fit the context and therefore might yield different results. Our measure of *social capital* appeared to be most problematic in that it was measured dichotomous, whereas other measures were not (see above). Despite several attempts to include this measure in different ways (e.g., by including individual items rather than sum scores), we could not find a way of scoring this scale that would improve its internal reliability or its relation to the other concepts we measured. In sum, we were unable to resolve this issue in the present research; in the future we would like to include other operationalisations of social capital to settle these issues. We believe such other measures should use Likert scales that leave more room for perceived connectedness. Adding such measure of capital would further enhance the comprehensiveness of our new integrative approach.

We also recognise that the bi-factor analysis approach has certain limitations and challenges (e.g. [64]) the most important of which, in our view, is that it can be challenging to interpret the meaning of general factors. However, we consider our theoretical rationale for a general factor of social relations strong enough to allow pursue this method in this case. After all, it makes sense for both measures of the quality and quantity of one’s social contacts to be related by underlying personal characteristics (whether one refers to them as capital or as sociability).

Perhaps the most important limitation in this research is that we suggest that there is scope for (and a need to) integrating the measures used into a comprehensive scale of *the perceived presence of others in one’s life*. Based on the current data collection, we can only speculate what such an instrument might look like. We would be hesitant to integrate items drawn from existing scales in a haphazard way: constructing a new scale would be a future challenge that should take a broader approach to sampling items in a considered way.

## Conclusion

The present research has shown that concepts on social relations and group affiliation, offer opportunities for integration. More importantly, it has proven that specifying particular *targets* or *subgroups*, might be essential in understanding how the structure of social relations influences particular outcomes that might benefit or harm individuals, for example in their health and wellbeing. We hope that our findings will stimulate researchers to critically evaluate their measures of social relations, and consider that the integration of concepts and measures across disciplines might be the way forward for this field.

## Supporting information

S1 Appendix (DOCX)
