# Supplementary material for: Social capital, identification and support: Scope for integration
Source: PLoS One. 2022 Apr 14;17(4):e0266499. doi: 10.1371/journal.pone.0266499 (PMC9009600; doi:10.1371/journal.pone.0266499)
Supplement: S1 Appendix — (DOCX) [file pone.0266499.s001.docx]

Appendix A

*Correlation matrix of study variables showing highlighted latent variables (Multiple Group Membership, Identification with Neighbourhood and Identification with Family/friends).*

| Variable | *M* | *SD* | 1 | 2 | 3 | 4 | 5 | 6 | 7 | 8 | 9 | 10 | 11 |
| --- | --- | --- | --- | --- | --- | --- | --- | --- | --- | --- | --- | --- | --- |
|  |  |  |  |  |  |  |  |  |  |  |  |  |  |
| 1. MGM 1 | 3.39 | 1.20 |  |  |  |  |  |  |  |  |  |  |  |
| 2. MGM 2 | 3.20 | 1.19 | .77** |  |  |  |  |  |  |  |  |  |  |
| 3. MGM 3 | 3.54 | 1.15 | .58** | .61** |  |  |  |  |  |  |  |  |  |
| 4. MGM 4 | 3.11 | 1.16 | .67** | .74** | .65** |  |  |  |  |  |  |  |  |
| 5. Ident neigh 1 | 3.76 | 0.94 | .38** | .41** | .37** | .42** |  |  |  |  |  |  |  |
| 6 Ident neigh 2 | 3.71 | 0.94 | .36** | .39** | .33** | .40** | .73** |  |  |  |  |  |  |
| 7. Ident neigh 3 | 3.26 | 1.11 | .34** | .36** | .31** | .41** | .57** | .64** |  |  |  |  |  |
| 8. Ident neigh 4 | 3.18 | 1.20 | .28** | .30** | .28** | .36** | .51** | .52** | .72** |  |  |  |  |
| 9. Ident fam/frie 1 | 4.20 | 0.79 | .22** | .23** | .24** | .24** | .28** | .30** | .24** | .23** |  |  |  |
| 10. Ident fam/frie 2 | 4.19 | 0.83 | .23** | .24** | .24** | .24** | .28** | .33** | .26** | .23** | .86** |  |  |
| 11. Ident fam/frie 3 | 3.66 | 1.15 | .21** | .22** | .22** | .26** | .24** | .24** | .43** | .40** | .57** | .60** |  |
| 12 Ident fam/frie 4 | 3.60 | 1.20 | .21** | .24** | .22** | .28** | .25** | .25** | .38** | .44** | .53** | .55** | .83** |

*Note.* *M* and *SD* are used to represent mean and standard deviation, respectively. * indicates *p* < .05. ** indicates *p* < .MGM 1-4 represent the 4 Multiple Group Membership scale items, Ident neigh 1-4 represent the 4 items of identification with the neighbourhood measure, Ident fam/frie 1-4 represent the 4 items of identification with family/friends measure. See Table 1 for corresponding item descriptions.

Appendix B

*Correlation matrix of study 2 variables*

| Variable | *M* | | *SD* | | 1 | | 2 | | 3 | | 4 | | 5 | | 6 | | 7 | | 8 | | 9 | | 10 | | 11 | | 12 | | 13 | | 14 | | 15 | | 16 | | 17 | | 18 | | 19 | | 20 | | 21 | | 22 | | 23 | 24 |
| --- | --- | --- | --- | --- | --- | --- | --- | --- | --- | --- | --- | --- | --- | --- | --- | --- | --- | --- | --- | --- | --- | --- | --- | --- | --- | --- | --- | --- | --- | --- | --- | --- | --- | --- | --- | --- | --- | --- | --- | --- | --- | --- | --- | --- | --- | --- | --- | --- | --- | --- |
| 1. MGM 1 | 3.09 | | 1.09 | |  | |  | |  | |  | |  | |  | |  | |  | |  | |  | |  | |  | |  | |  | |  | |  | |  | |  | |  | |  | |  | |  | |  |  |
| 2. MGM 2 | 2.92 | | 1.09 | | .86** | |  | |  | |  | |  | |  | |  | |  | |  | |  | |  | |  | |  | |  | |  | |  | |  | |  | |  | |  | |  | |  | |  |  |
| 3. MGM 3 | 3.22 | | 0.99 | | .70** | | .70** | |  | |  | |  | |  | |  | |  | |  | |  | |  | |  | |  | |  | |  | |  | |  | |  | |  | |  | |  | |  | |  |  |
| 4. MGM 4 | 2.79 | | 0.99 | | .79** | | .81** | | .71** | |  | |  | |  | |  | |  | |  | |  | |  | |  | |  | |  | |  | |  | |  | |  | |  | |  | |  | |  | |  |  |
| 5. Support neigh | 3.18 | | 0.92 | | .42** | | .43** | | .45** | | .47** | |  | |  | |  | |  | |  | |  | |  | |  | |  | |  | |  | |  | |  | |  | |  | |  | |  | |  | |  |  |
| 6. Ident neigh | 3.39 | | 0.86 | | .37** | | .39** | | .37** | | .41** | | .67** | |  | |  | |  | |  | |  | |  | |  | |  | |  | |  | |  | |  | |  | |  | |  | |  | |  | |  |  |
| 7. Ident neigh 1 | 2.92 | | 0.90 | | .31** | | .31** | | .27** | | .34** | | .47** | | .62** | |  | |  | |  | |  | |  | |  | |  | |  | |  | |  | |  | |  | |  | |  | |  | |  | |  |  |
| 8. Ident neigh 2 | 3.42 | | 0.83 | | .34** | | .35** | | .31** | | .36** | | .57** | | .74** | | .64** | |  | |  | |  | |  | |  | |  | |  | |  | |  | |  | |  | |  | |  | |  | |  | |  |  |
| 9. Ident neigh 3 | 3.49 | | 0.83 | | .31** | | .33** | | .30** | | .33** | | .54** | | .71** | | .60** | | .75** | |  | |  | |  | |  | |  | |  | |  | |  | |  | |  | |  | |  | |  | |  | |  |  |
| 10. Ident neigh 4 | 2.86 | | 0.95 | | .28** | | .30** | | .25** | | .34** | | .47** | | .58** | | .62** | | .59** | | .63** | |  | |  | |  | |  | |  | |  | |  | |  | |  | |  | |  | |  | |  | |  |  |
| 11. Ident fam/frie | 4.12 | | 0.72 | | .21** | | .20** | | .23** | | .23** | | .26** | | .29** | | .21** | | .30** | | .30** | | .18** | |  | |  | |  | |  | |  | |  | |  | |  | |  | |  | |  | |  | |  |  |
| 12. Support fam/frie | 4.07 | | 0.73 | | .19** | | .18** | | .23** | | .21** | | .31** | | .25** | | .18** | | .25** | | .25** | | .15** | | .78** | |  | |  | |  | |  | |  | |  | |  | |  | |  | |  | |  | |  |  |
| 13. Support SP 1 | 4.14 | | 0.96 | | .15** | | .13** | | .17** | | .14** | | .17** | | .10** | | .09** | | .12** | | .11** | | .03 | | .26** | | .32** | |  | |  | |  | |  | |  | |  | |  | |  | |  | |  | |  |  |
| 14. Support SP 2 | 4.30 | | 0.85 | | .15** | | .13** | | .17** | | .14** | | .19** | | .14** | | .10** | | .13** | | .12** | | .04 | | .27** | | .34** | | .76** | |  | |  | |  | |  | |  | |  | |  | |  | |  | |  |  |
| 15. Support family 1 | 4.11 | | 0.83 | | .15** | | .15** | | .18** | | .17** | | .22** | | .18** | | .13** | | .18** | | .18** | | .12** | | .32** | | .39** | | .35** | | .35** | |  | |  | |  | |  | |  | |  | |  | |  | |  |  |
| 16. Support family 2 | 4.06 | | 0.88 | | .15** | | .15** | | .17** | | .18** | | .24** | | .20** | | .16** | | .19** | | .19** | | .15** | | .33** | | .42** | | .34** | | .35** | | .84** | |  | |  | |  | |  | |  | |  | |  | |  |  |
| 17. Support SP 3 | 4.14 | | 0.96 | | .12** | | .11** | | .16** | | .15** | | .19** | | .12** | | .10** | | .13** | | .12** | | .05** | | .26** | | .34** | | .71** | | .75** | | .36** | | .39** | |  | |  | |  | |  | |  | |  | |  |  |
| 18. Support family 3 | 3.98 | | 0.79 | | .27** | | .27** | | .34** | | .32** | | .36** | | .27** | | .17** | | .26** | | .23** | | .14** | | .28** | | .36** | | .44** | | .48** | | .46** | | .47** | | .50** | |  | |  | |  | |  | |  | |  |  |
| 19. Support family 4 | 4.05 | | 0.76 | | .29** | | .28** | | .34** | | .32** | | .36** | | .29** | | .18** | | .26** | | .25** | | .14** | | .27** | | .34** | | .40** | | .44** | | .39** | | .41** | | .44** | | .85** | |  | |  | |  | |  | |  |  |
| 20. Support friends 1 | 3.97 | | 0.90 | | .15** | | .16** | | .17** | | .20** | | .25** | | .21** | | .17** | | .20** | | .20** | | .15** | | .33** | | .40** | | .29** | | .31** | | .73** | | .77** | | .33** | | .43** | | .40** | |  | |  | |  | |  |  |
| 21. Support friends 2 | 4.08 | | 0.81 | | .30** | | .29** | | .38** | | .34** | | .35** | | .30** | | .18** | | .27** | | .25** | | .14** | | .30** | | .35** | | .40** | | .45** | | .36** | | .39** | | .45** | | .74** | | .78** | | .41** | |  | |  | |  |  |
| 22. Support SP 4 | 4.34 | | 0.85 | | .14** | | .12** | | .17** | | .15** | | .19** | | .15** | | .10** | | .15** | | .13** | | .06** | | .27** | | .34** | | .59** | | .70** | | .35** | | .37** | | .68** | | .42** | | .40** | | .31** | | .43** | |  | |  |  |
| 23. Support friends 3 | 3.95 | | 0.89 | | .17** | | .15** | | .17** | | .19** | | .25** | | .19** | | .17** | | .19** | | .17** | | .14** | | .32** | | .40** | | .31** | | .32** | | .75** | | .75** | | .35** | | .45** | | .41** | | .76** | | .39** | | .33** | |  |  |
| 24. Support friends 4 | 4.01 | | 0.81 | | .30** | | .28** | | .37** | | .34** | | .36** | | .29** | | .20** | | .26** | | .24** | | .14** | | .29** | | .36** | | .42** | | .45** | | .36** | | .40** | | .46** | | .75** | | .77** | | .42** | | .82** | | .41** | | .43** |  |
| 25. Soc. capital | 7.54 | | 2.34 | | .33** | | .35** | | .28** | | .32** | | .37** | | .36** | | .24** | | .33** | | .33** | | .23** | | .17** | | .16** | | .11** | | .11** | | .17** | | .15** | | .09** | | .22** | | .21** | | .13** | | .20** | | .08** | | .13** | .21** |
|  |  |  | |  | |  | |  | |  | |  | |  | |  | |  | |  | |  | |  | |  | |  | |  | |  | |  | |  | |  | |  | |  | |  | |  | |  | |  |

*Note. M* and *SD* are used to represent mean and standard deviation, respectively. * indicates *p* < .05. ** indicates *p* < .MGM 1-4 represent the 4 Multiple Group Membership scale items, Support neigh represents the single item social support from neighbourhood measure, Ident neigh represents the single item identification with neighbourhood measure, Ident neigh 1-4 represent the 4 items of identification with the neighbourhood measure, Ident fam/frie represents the single item identification with family/friends measure, Support fam/frie represents the single item social support from family/fiends measure, Support SP 1-4 represent items of measuring social support from “special person”, Support family 1-4 represent items of measuring social support from family, Support friends 1-4 represent items of measuring social support from friends, Soc. capital represents the sum score measure for social capital. See Table 1 for corresponding item descriptions.

Appendix C

*Correlation matrix of study 2 variables, with single items social capital scale*

| Variable | MGM 1 | MGM 2 | MGM 3 | MGM 4 | Support neigh | Ident neigh | Ident neigh 1 | Ident neigh 2 | Ident neigh 3 | Ident neigh 4 | Ident fam/frie | Support fam/frie | Support SP 1 | Support SP 2 | Support family 1 | Support family 2 | Support SP 3 | Support family 3 | Support family 4 | Support friends 1 | Support friends 2 | Support SP 4 | Support friends 3 | Support friends 4 |
| --- | --- | --- | --- | --- | --- | --- | --- | --- | --- | --- | --- | --- | --- | --- | --- | --- | --- | --- | --- | --- | --- | --- | --- | --- |
| SC 1 | 0.22 | 0.27 | 0.16 | 0.21 | 0.24 | 0.28 | 0.14 | 0.20 | 0.27 | 0.26 | 0.24 | 0.05 | 0.04 | 0.00 | -0.03 | -0.03 | 0.01 | 0.02 | -0.05 | 0.03 | 0.03 | 0.00 | 0.02 | -0.02 |
| SC 2 | 0.16 | 0.19 | 0.12 | 0.15 | 0.11 | 0.14 | 0.09 | 0.10 | 0.12 | 0.13 | 0.10 | 0.05 | 0.00 | 0.02 | -0.03 | 0.00 | 0.03 | 0.03 | -0.02 | 0.00 | 0.00 | 0.04 | 0.02 | 0.03 |
| SC 3 | 0.24 | 0.25 | 0.18 | 0.21 | 0.09 | 0.08 | -0.02 | 0.07 | 0.06 | 0.07 | 0.07 | 0.01 | 0.01 | -0.04 | 0.03 | 0.02 | 0.02 | 0.03 | -0.01 | 0.06 | 0.08 | 0.02 | 0.07 | -0.01 |
| SC 4 | 0.10 | 0.11 | 0.07 | 0.08 | 0.06 | 0.06 | 0.05 | 0.07 | 0.04 | 0.06 | 0.07 | 0.04 | 0.03 | 0.03 | -0.01 | 0.01 | -0.01 | -0.01 | 0.02 | 0.05 | 0.04 | -0.01 | 0.03 | 0.01 |
| SC 5 | 0.09 | 0.11 | 0.07 | 0.07 | 0.01 | 0.06 | 0.06 | 0.06 | 0.04 | 0.06 | 0.05 | 0.00 | -0.01 | -0.01 | -0.04 | -0.04 | 0.00 | -0.04 | -0.01 | -0.01 | -0.01 | -0.02 | -0.02 | -0.03 |
| SC 6 | 0.08 | 0.08 | 0.07 | 0.07 | 0.03 | 0.02 | 0.05 | 0.03 | 0.05 | 0.03 | 0.02 | 0.04 | 0.02 | 0.04 | 0.02 | 0.01 | 0.02 | 0.01 | 0.01 | 0.00 | 0.02 | -0.01 | 0.02 | 0.00 |
| SC 7 | 0.14 | 0.16 | 0.10 | 0.16 | 0.14 | 0.10 | 0.09 | 0.07 | 0.11 | 0.10 | 0.09 | 0.10 | 0.07 | 0.03 | 0.05 | 0.04 | 0.05 | 0.05 | 0.05 | 0.09 | 0.09 | 0.05 | 0.09 | 0.03 |
| SC 8 | 0.06 | 0.04 | 0.07 | 0.05 | 0.12 | 0.06 | 0.06 | 0.05 | 0.06 | 0.06 | 0.01 | 0.13 | 0.15 | 0.13 | 0.18 | 0.17 | 0.28 | 0.25 | 0.15 | 0.18 | 0.15 | 0.21 | 0.17 | 0.10 |
| SC 9 | 0.08 | 0.07 | 0.08 | 0.07 | 0.28 | 0.21 | 0.14 | 0.12 | 0.17 | 0.18 | 0.10 | 0.09 | 0.13 | 0.08 | 0.11 | 0.11 | 0.13 | 0.11 | 0.11 | 0.16 | 0.13 | 0.10 | 0.15 | 0.09 |
| SC 10 | 0.16 | 0.12 | 0.18 | 0.15 | 0.17 | 0.09 | 0.06 | 0.05 | 0.09 | 0.08 | 0.00 | 0.13 | 0.14 | 0.11 | 0.19 | 0.19 | 0.14 | 0.12 | 0.19 | 0.30 | 0.29 | 0.10 | 0.33 | 0.12 |
| SC 11 | 0.08 | 0.09 | 0.07 | 0.08 | 0.04 | 0.03 | 0.02 | 0.01 | 0.04 | 0.05 | 0.03 | 0.02 | 0.04 | 0.01 | 0.00 | 0.01 | 0.04 | 0.02 | 0.00 | 0.02 | 0.05 | 0.03 | 0.02 | 0.00 |
| SC 12 | 0.07 | 0.08 | 0.07 | 0.08 | 0.08 | 0.07 | 0.06 | 0.03 | 0.06 | 0.05 | 0.01 | 0.05 | 0.03 | 0.03 | 0.02 | 0.03 | 0.03 | 0.01 | 0.01 | 0.05 | 0.04 | 0.01 | 0.01 | 0.01 |
| SC 13 | 0.13 | 0.15 | 0.09 | 0.14 | 0.09 | 0.08 | 0.06 | 0.05 | 0.07 | 0.07 | 0.07 | 0.09 | 0.05 | 0.05 | 0.04 | 0.03 | 0.04 | 0.04 | 0.04 | 0.05 | 0.04 | 0.02 | 0.04 | 0.00 |
| SC 14 | 0.09 | 0.11 | 0.05 | 0.09 | 0.09 | 0.09 | 0.06 | 0.06 | 0.08 | 0.09 | 0.07 | 0.06 | 0.03 | 0.03 | 0.00 | 0.00 | 0.05 | 0.06 | 0.00 | 0.04 | 0.05 | 0.04 | 0.03 | 0.01 |
| SC 15 | 0.16 | 0.17 | 0.15 | 0.16 | 0.17 | 0.17 | 0.16 | 0.12 | 0.16 | 0.14 | 0.10 | 0.04 | 0.04 | 0.02 | 0.02 | 0.01 | 0.04 | 0.04 | 0.01 | 0.06 | 0.04 | 0.03 | 0.03 | -0.01 |
| SC 16 | 0.05 | 0.07 | 0.05 | 0.05 | 0.08 | 0.09 | 0.10 | 0.04 | 0.09 | 0.08 | 0.05 | 0.00 | -0.01 | -0.01 | -0.01 | -0.02 | -0.01 | -0.04 | -0.01 | -0.02 | -0.02 | -0.03 | -0.02 | -0.01 |
| SC 17 | 0.04 | 0.05 | 0.04 | 0.04 | 0.15 | 0.18 | 0.07 | 0.13 | 0.16 | 0.20 | 0.13 | 0.04 | 0.02 | -0.02 | -0.01 | 0.00 | 0.06 | 0.06 | 0.00 | 0.05 | 0.05 | 0.05 | 0.04 | 0.03 |
| SC 18 | 0.08 | 0.08 | 0.06 | 0.09 | 0.18 | 0.22 | 0.07 | 0.15 | 0.21 | 0.24 | 0.14 | 0.07 | 0.03 | 0.00 | 0.02 | 0.02 | 0.07 | 0.07 | 0.00 | 0.07 | 0.06 | 0.07 | 0.05 | 0.01 |
| SC 19 | 0.15 | 0.16 | 0.11 | 0.16 | 0.32 | 0.42 | 0.17 | 0.27 | 0.39 | 0.40 | 0.29 | 0.10 | 0.10 | -0.02 | 0.02 | 0.05 | 0.09 | 0.12 | 0.02 | 0.11 | 0.13 | 0.11 | 0.10 | 0.04 |
| SC 20 | -0.06 | -0.06 | -0.05 | -0.05 | -0.13 | -0.15 | -0.03 | -0.10 | -0.13 | -0.12 | -0.08 | -0.10 | -0.08 | -0.02 | -0.03 | -0.04 | -0.07 | -0.07 | -0.02 | -0.07 | -0.06 | -0.10 | -0.04 | -0.03 |
|  |  |  |  |  |  |  |  |  |  |  |  |  |  |  |  |  |  |  |  |  |  |  |  |  |

Appendix D

*Tetrachoric Correlation matrix of single items social capital scale*

| Variable | SC 1 | SC 2 | SC 3 | SC 4 | SC 5 | SC 6 | SC 7 | SC 8 | SC 9 | SC 10 | SC 11 | SC 12 | SC 13 | SC 14 | SC 15 | SC 16 | SC 17 | SC 18 | SC 19 | SC 20 |
| --- | --- | --- | --- | --- | --- | --- | --- | --- | --- | --- | --- | --- | --- | --- | --- | --- | --- | --- | --- | --- |
| SC 1 | 1 |  |  |  |  |  |  |  |  |  |  |  |  |  |  |  |  |  |  |  |
| SC 2 | 0.12 |  |  |  |  |  |  |  |  |  |  |  |  |  |  |  |  |  |  |  |
| SC 3 | 0.10 | -0.02 |  |  |  |  |  |  |  |  |  |  |  |  |  |  |  |  |  |  |
| SC 4 | 0.07 | 0.01 | 0.05 |  |  |  |  |  |  |  |  |  |  |  |  |  |  |  |  |  |
| SC 5 | 0.04 | 0.13 | 0.03 | 0.06 |  |  |  |  |  |  |  |  |  |  |  |  |  |  |  |  |
| SC 6 | 0.08 | 0.02 | 0.01 | 0.08 | 0.05 |  |  |  |  |  |  |  |  |  |  |  |  |  |  |  |
| SC 7 | 0.14 | 0.25 | 0.06 | 0.08 | 0.07 | 0.09 |  |  |  |  |  |  |  |  |  |  |  |  |  |  |
| SC 8 | 0.03 | 0.04 | 0.01 | 0.01 | -0.04 | 0.01 | 0.09 |  |  |  |  |  |  |  |  |  |  |  |  |  |
| SC 9 | 0.11 | -0.02 | 0.01 | 0.04 | 0.00 | 0.05 | 0.15 | 0.19 |  |  |  |  |  |  |  |  |  |  |  |  |
| SC 10 | 0.05 | 0.02 | 0.07 | 0.04 | -0.02 | 0.06 | 0.17 | 0.23 | 0.07 |  |  |  |  |  |  |  |  |  |  |  |
| SC 11 | 0.07 | 0.11 | 0.02 | 0.00 | 0.25 | 0.05 | 0.08 | 0.04 | 0.07 | 0.06 |  |  |  |  |  |  |  |  |  |  |
| SC 12 | 0.09 | 0.04 | 0.05 | 0.03 | 0.11 | 0.08 | 0.13 | 0.10 | 0.15 | 0.11 | 0.28 |  |  |  |  |  |  |  |  |  |
| SC 13 | 0.09 | 0.11 | 0.04 | 0.03 | 0.08 | 0.06 | 0.21 | 0.10 | 0.10 | 0.13 | 0.14 | 0.22 |  |  |  |  |  |  |  |  |
| SC 14 | 0.06 | 0.55 | 0.00 | 0.02 | 0.08 | 0.01 | 0.09 | 0.09 | 0.08 | 0.13 | 0.15 | 0.07 | 0.22 |  |  |  |  |  |  |  |
| SC 15 | 0.26 | -0.02 | 0.04 | 0.07 | 0.07 | 0.12 | 0.12 | 0.09 | 0.16 | 0.12 | 0.04 | 0.15 | 0.08 | 0.04 |  |  |  |  |  |  |
| SC 16 | 0.09 | 0.00 | 0.01 | 0.01 | 0.06 | 0.13 | 0.10 | 0.07 | 0.09 | 0.09 | 0.06 | 0.13 | 0.11 | 0.06 | 0.47 |  |  |  |  |  |
| SC 17 | 0.02 | 0.02 | 0.03 | -0.01 | 0.03 | 0.00 | 0.01 | 0.01 | 0.06 | 0.02 | 0.02 | 0.03 | 0.00 | 0.04 | 0.02 | 0.00 |  |  |  |  |
| SC 18 | 0.03 | 0.03 | 0.02 | -0.02 | 0.01 | -0.02 | 0.02 | 0.04 | 0.07 | 0.02 | 0.02 | 0.00 | 0.00 | 0.01 | 0.02 | -0.01 | 0.14 |  |  |  |
| SC 19 | 0.08 | 0.05 | 0.04 | 0.03 | 0.01 | 0.00 | 0.02 | -0.02 | 0.13 | 0.01 | -0.01 | 0.02 | 0.01 | 0.03 | 0.09 | 0.05 | 0.24 | 0.11 |  |  |
| SC 20 | -0.02 | -0.02 | -0.01 | 0.01 | 0.00 | 0.03 | 0.00 | -0.02 | -0.01 | -0.01 | -0.01 | -0.02 | 0.04 | -0.03 | 0.01 | 0.04 | -0.04 | -0.30 | -0.17 | 1 |
|  |  |  |  |  |  |  |  |  |  |  |  |  |  |  |  |  |  |  |  |  |
